# *HOXA7* Expression Is an Independent Prognostic Biomarker in Esophageal Squamous Cell Carcinoma

**DOI:** 10.3390/genes15111430

**Published:** 2024-11-01

**Authors:** Jennifer Vieira Gomes, Pedro Nicolau-Neto, Júlia Nascimento de Almeida, Lilian Brewer Lisboa, Paulo Thiago de Souza-Santos, Luis Felipe Ribeiro-Pinto, Sheila Coelho Soares-Lima, Tatiana de Almeida Simão

**Affiliations:** 1 Laboratório de Toxicologia e Biologia Molecular, Departamento de Bioquímica, Universidade do Estado do Rio de Janeiro (UERJ), Rio de Janeiro 20550-013, RJ, Brazil; vieira.jb@hotmail.com (J.V.G.); juh.na@hotmail.com (J.N.d.A.); lilian.brewer06@gmail.com (L.B.L.); lfrpinto@inca.gov.br (L.F.R.-P.); 2 Programa de Carcinogênese Molecular, Instituto Nacional de Câncer (INCA), Rio de Janeiro 20230-130, RJ, Brazil; pedronicolau.n@gmail.com; 3 Beneficência Portuguesa de São Paulo, São Paulo 01323-001, SP, Brazil; pthiagoss@gmail.com; 4 Programa de Pesquisa Clínica e Desenvolvimento Tecnológico, Instituto Nacional de Câncer (INCA), Rio de Janeiro 20230-130, RJ, Brazil; sheilacoelho@gmail.com

**Keywords:** *HOX* genes, *HOXA7*, esophageal squamous cell carcinoma, prognostic biomarker

## Abstract

**Background/Objectives**: Homeobox (*HOX*) genes encode conserved transcription factors essential for tissue and organ development and cellular differentiation. In humans, these genes are organized into four clusters: HOXA, HOXB, HOXC, and HOXD. While *HOX* genes have been extensively studied in cancer biology, their roles in esophageal squamous cell carcinoma (ESCC) remain poorly understood. Given the increasing incidence and high mortality rate of ESCC, exploring the molecular drivers of this tumor is urgent. **Methods**: Therefore, this study investigated the mutational landscape and expression profiles of *HOX* genes in ESCC and their differentially expressed targets using ESCC data from The Cancer Genome Atlas (TCGA) and two independent transcriptome datasets. **Results**: We found that copy number alterations and single nucleotide variations were rare, while seven *HOX* genes (*HOXA2*, *HOXA7*, *HOXB13*, *HOXC9*, *HOXC10*, *HOXC13*, and *HOXD10*) were significantly differentially expressed in ESCC compared to paired non-malignant mucosa. Further analysis identified 776 potential *HOX* target genes differentially expressed in ESCC, many of which are involved in critical cancer pathways such as PI3K-AKT, cell cycle regulation, and epithelial–mesenchymal transition (EMT). The *HOXA7* overexpression was associated with poor overall survival rates in ESCC. This finding opens new possibilities for targeted therapies, offering hope for improved patient outcomes. **Conclusions**: Thus, this study underscored the pivotal role of *HOX* gene dysregulation in ESCC and classified *HOXA7* as a potential prognostic biomarker in this tumor.

## 1. Introduction

Homeobox (*HOX*) genes are a group of major transcription factors, distinguished by a highly conserved 61-amino-acid helix-turn-helix DNA binding homeodomain, preserved throughout animal evolution. In humans, there are 39 *HOX* genes organized into four clusters located on different chromosomes: HOXA (7p15), HOXB (17q21), HOXC (12q13), and HOXD (2q31) [1]. *HOX* proteins can function as monomers or homodimers to regulate the transcription of downstream targets. Sometimes, they form heterodimers or heterotrimers with members of the TALE (three amino acid loop extension) family of cofactors, depending on the specific cell type [2]. The coordinated expression of *HOX* genes is essential for defining the embryonic anterior–posterior body axis and determining temporospatial limb development, tissue and organ formation, and cellular differentiation [3,4].

While the role of *HOX* genes in cancer is becoming more evident, it is still not fully understood. In certain cancers, specific *HOX* genes that generally suppress tumors are silenced, whereas, in other contexts, *HOX* genes are overexpressed and contribute to oncogenic processes [2]. *HOXB13* is a classic example of this deregulation, displaying a dual role depending on tissue type. For instance, while *HOXB13* is essential for proper prostate development and differentiation [5], its overexpression in breast cancer is linked to greater invasiveness by downregulating the estrogen receptor α and upregulating interleukin-6 expression [6]. *HOX* gene deregulation has been reported in various cancers, including leukemia, breast, ovarian, prostate, gastric, and esophageal cancers [7,8,9,10,11,12,13,14]. Previous studies have highlighted the role of HOXA in cellular processes, particularly within the context of innate immune cell activation [12,14]. The roles of HOXB, HOXC, and HOXD in esophageal squamous cell carcinoma (ESCC) are not yet fully elucidated. Research suggests that specific genes within these clusters contribute to tumor progression, like *HOXB13*, *HOXC10*, and *HOXD13* [14]. Esophageal cancer (EC) is the seventh most common cancer type and the sixth leading cause of cancer-related deaths worldwide among men. Alarmingly, the incidence of EC is projected to increase 1.8-fold in both sexes by 2050 [15]. There are two primary histological subtypes of EC: esophageal adenocarcinoma and ESCC, with ESCC accounting for 85% of all EC cases. Over the past two decades, treatment advancements have included a combination of neoadjuvant chemotherapy, radiotherapy, and surgery [16]. However, despite these efforts, the survival rates for EC remain poor. The underlying molecular mechanisms driving ESCC initiation, promotion, and progression are still not fully understood, contributing to the bleak patient outcomes. Therefore, research to identify biomarkers for improved ESCC diagnosis, prognosis, and treatment is critical to changing this scenario [17].

This study investigated the mutational landscape and gene expression profiles of *HOX* genes in ESCC. Through comparative analysis between tumor tissues and their paired nonmalignant mucosa, we identified *HOXA7* overexpression as a critical prognostic biomarker associated with poor outcomes in ESCC patients.

## 2. Materials and Methods

### 2.1. Analysis of HOX Gene Expression Using Publicly Available Esophageal

Two transcriptome datasets of tumor and matched non-malignant surrounding mucosa (NMSM) were utilized to investigate the *HOX* gene expression profile. These included our previously published transcriptome analysis dataset (GSE75241) [18] and the dataset by Li and colleagues (GSE53625) [19]. *HOX* gene expression in ESCC was quantified by calculating gene expression values in array units.

Gene expression analyses were conducted in the R environment, with differential expression assessed using the Limma package from the Bioconductor project. Differentially expressed genes (DEGs) were identified based on an adjusted *p*-value < 0.05 and a fold-change threshold of |1.5| [20,21].

### 2.2. Evaluation of Somatic Alterations in HOX Genes in the TCGA Data

The frequency of copy number alterations (CNAs) and single nucleotide variants (SNVs) in *HOX* genes in ESCC was assessed using The Cancer Genome Atlas (TCGA) dataset by applying The Genomic Identification of Significant Targets in Cancer (GISTIC) 2.0 protocol to whole exome sequencing [22].

### 2.3. Snap-Frozen Human Tissue Samples

Forty-one ESCC patients treated between 2012 and 2015 at the Instituto Nacional de Câncer (INCA, Rio de Janeiro, Brazil) were enrolled in this study. Snap-frozen tumor and matched non-malignant surrounding mucosa (NMSM) samples were collected by endoscopy before chemotherapy or radiotherapy. These samples were stored at the INCA Tumor Bank (Banco Nacional de Tumores e DNA, BNT) until RNA extraction. The INCA Ethics Committee approved the study (Protocol 116/11). Patient characteristics are summarized in Table 1.

### 2.4. Evaluation of Gene Expression by Quantitative PCR (qPCR)

Total RNA was extracted from snap-frozen biopsies using the RNeasy mini kit (Qiagen) following the manufacturer’s protocol. Complementary DNA (cDNA) was synthesized using SuperScript II (Invitrogen, CA, USA) and random primers (Invitrogen, California, USA), according to the manufacturer’s instructions.

*HOX* expression was assessed by qPCR using 41 paired ESCC and NMSM samples. qPCR was performed with the Quantifast SYBR Green PCR kit (Qiagen), Valencia, CA, USA) in a Rotor-Gene 6000 thermal cycler (Qiagen). Gene expression quantification was conducted as previously described [23]. Specific oligonucleotide sequences are listed in Supplementary Table S1.

**Figure 1:**
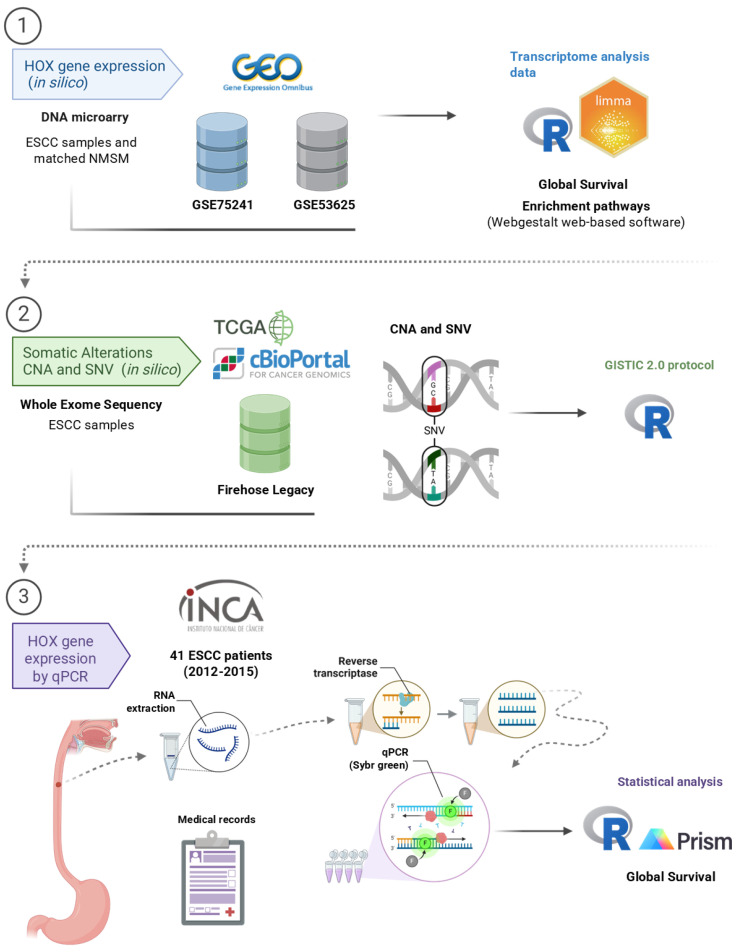
Summary schematic of the principal methodologies and sample datasets used. ESCC: esophageal squamous cell carcinoma; matched non-malignant surrounding mucosa; CNA; copy number alteration; SNV: single nucleotide variant: GISTIC: The Genomic Identification of Significant Targets in Cancer (created in BioRender).

A schematic diagram (Figure 1) was created to highlight the primary methodological information addressed in this study and facilitate understanding of the methods and sample sets used.

### 2.5. Identification of HOX Targets and Gene-Set Enrichment Analyses

The Transcriptome Factor Target Gene database identified the putative transcriptional targets of selected *HOX* genes [24]. Next, we evaluated whether these targets were among the 1368 DEGs identified in ESCC from our previously published study (GSE75241) [18]. The differentially expressed *HOX* targets were then subjected to gene-set enrichment analysis using the WEB-based GEne SeT AnaLysis Toolkit 2024 (WebGestalt) [25], with pathways evaluated using the Kyoto Encyclopedia of Genes and Genomes (KEGG) and Hallmark_50 databases. Pathways were considered enriched if the adjusted *p*-value was <0.05.

### 2.6. Statistical Analyses

The Kolmogorov–Smirnov test was used to assess the normality of continuous data, followed by paired *t*-tests, Wilcoxon matched-pairs signed-rank tests, unpaired *t*-tests, or Mann–Whitney U tests, as appropriate. These analyses were performed using GraphPad Prism 8 software. The Kaplan–Meier method and log-rank test were applied to estimate the impact of individual variables on overall survival (OS). Variables with a *p*-value < 0.05 were selected for multivariate analysis. Cox regression analysis was performed using the stepwise backward method [26]. Survival analyses were conducted in the R environment using the survival package [27]. Gene expression cut-offs were determined using the best-performing threshold [28], with at least 20% of samples in one of the comparison groups. For Brazilian patients, samples were classified as high and low *HOXA7* expression with an expression cut-off of 0.00256 relative to *GAPDH*. For the GSE53625 dataset, the *HOXA7* expression cut-off was 13.0277 array units.

## 3. Results

### 3.1. Mutational Profile of HOX Genes

The frequency of somatic genetic alterations in *HOX* genes in ESCC was evaluated in the TCGA dataset. Among ninety-five ESCC samples, six (6.3%) exhibited amplification, and one showed deletion in the *HOXA* locus 7p15.2. One sample showed amplification in the HOXB locus 17q21.3, and one showed amplification of *HOXD locus 2q31*. In addition, missense mutations occurred at a low frequency in ESCC samples (≤1%) (Supplementary Figure S1).

### 3.2. HOX Genes’ Expression Profile

The expression profile of *HOX* genes in ESCC was assessed using our previously published transcriptome dataset (GSE75241), comparing paired ESCC and NMSM samples. We identified seven *HOX* genes with differential expression in ESCC, six of which were upregulated (*HOXA7*, *HOXB13*, *HOXC9*, *HOXC10*, *HOXC13*, and *HOXD10*) and one downregulated (*HOXA2*) (Table 2). The differential expression of these seven *HOX* genes was further validated using the independent dataset GSE53625 (Table 2).

### 3.3. Targets of ESCC-Overexpressed HOX Genes Are Associated with Enriched Signaling Pathways and Cellular Processes

**Figure 2:**
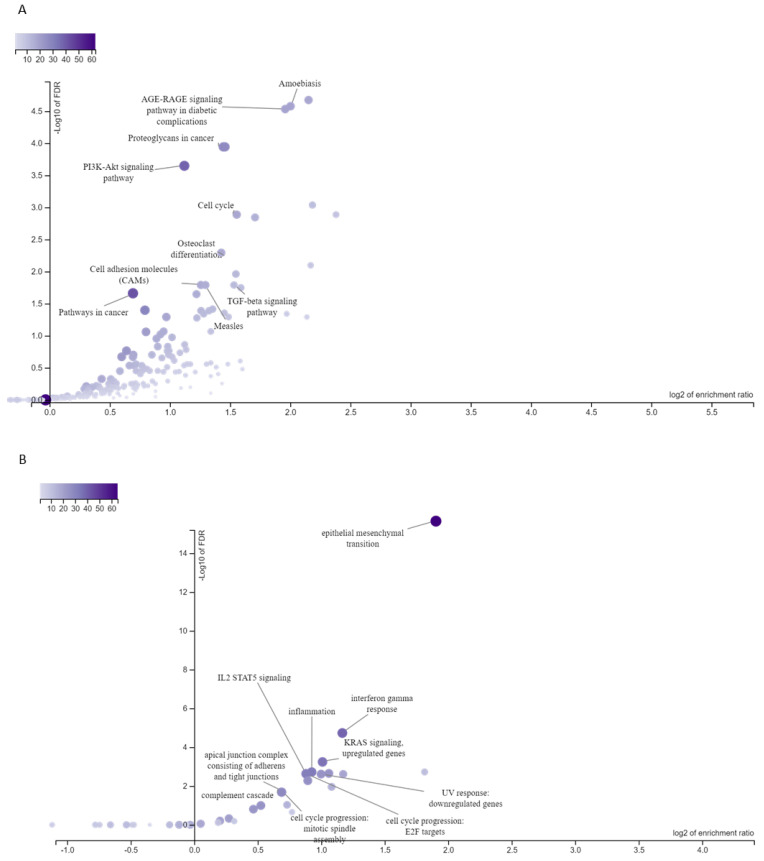
Enrichment analyses of differentially expressed *HOX* gene targets. Enrichment analyses using the potential targets of differentially expressed *HOX* genes in esophageal squamous cell carcinoma (ESCC) highlighted significant signaling pathways in KEGG (**A**) and Hallmarks (**B**) databases. Shades of blue indicate the number of genes observed in each signaling pathway. The darker blue shows more observed genes in the signaling pathway.

Initially, we identified potential transcriptional targets of *HOXA2*, *HOXA7*, *HOXB13*, *HOXC9*, *HOXC10*, *HOXC13*, and *HOXD10* using the Transcription Factor Target Gene database. We then integrated these data with our previously published ESCC transcriptome dataset (GSE75241) to identify which *HOX* gene targets were differentially expressed (DEG) in ESCC. Of 1368 DEGs identified in ESCC, 776 were potential *HOX* gene targets (Supplementary Table S2). A thorough gene-set enrichment analysis of these 776 putative *HOX* targets using the KEGG database revealed significant enrichment in pathways related to PI3K-AKT signaling, cell cycle regulation, cell adhesion molecules, and cancer-associated signaling pathways (Figure 2A). A detailed analysis using the Cellular Hallmarks database highlighted the enrichment of key cancer hallmarks, including inflammation (inflammatory response) and cellular plasticity (epithelial–mesenchymal transition) (Figure 2B). We applied the same methodology to analyze *HOXA7* targets and found that 223 of the 776 putative *HOX* targets were identified as targets of this gene (Supplementary Table S3). The KEGG pathway enrichment analysis of these putative targets is strongly associated with cell adhesion molecule pathways. In contrast, the Cellular Hallmarks database analysis highlighted significant enrichment in epithelial–mesenchymal transition, reflecting critical aspects of cellular plasticity and tumor progression.

### 3.4. Association Analyses Between ESCC-Overexpressed HOX Genes and Clinicopathological Features

Association analyses between the expression of *HOXA2*, *HOXA7*, *HOXB13*, *HOXC9*, *HOXC10*, *HOXC13*, and *HOXD10* and clinicopathological features were conducted with the GSE53625 dataset. *HOXB13* was overexpressed in older patients (>60 years) relative to younger patients. The expression of *HOXD10* was higher in tumors of males relative to female patients and in tumors of patients with a history of alcohol drinking. Furthermore, ESCC from patients with tobacco smoking habits and with poorly tumor grades showed *HOXC9* overexpression relative to never-smokers and patients with other tumor grades, respectively (Table 3).

**Figure 3:**
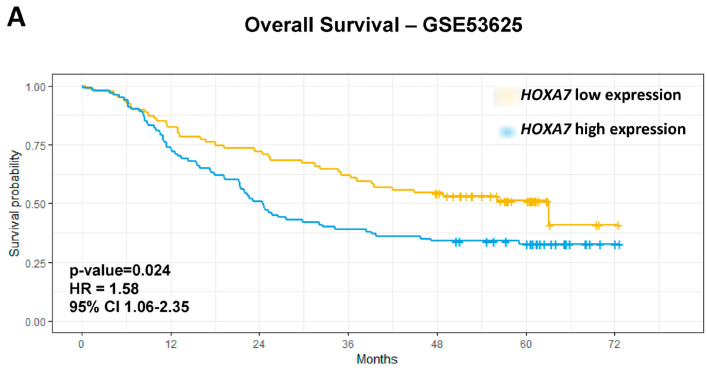

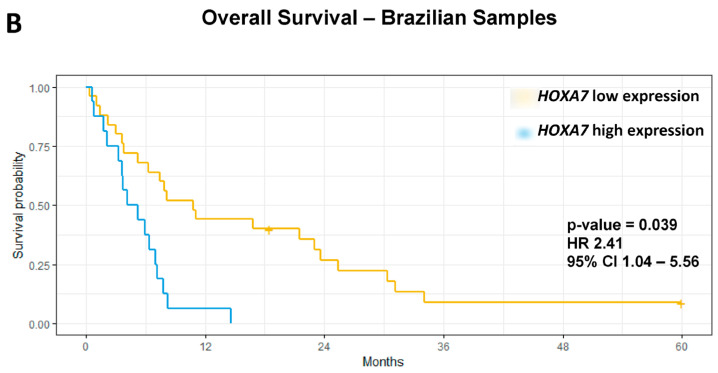
*HOXA7* overexpression is an independent biomarker of worse prognosis in ESCC. Patients with esophageal squamous cell carcinoma (ESCC) expressing high *HOXA7* gene levels showed worse survival rates than patients with ESCC expressing low *HOXA7* levels in the GSE53625 dataset ((**A**) *p* = 0.024; HR = 1.58; 95% CI 1.06–2.35) and the Brazilian Samples ((**B**) *p*-value = 0.039, HR 2.41, 95% CI 1.04–5.56). Yellow lines—samples with low *HOXA7* expression; Blue lines—samples with high *HOXA7* expression.

Regarding overall survival, univariate analyses showed that age > 60 years (*p* = 0.028), late-stage (III/IV) (*p* = 0.0002), high-grade tumors (G2/G3) (*p* = 0.048), *HOXA2* low expression (*p* = 0.032), *HOXA7* overexpression (*p* = 0.013), *HOXB13* overexpression (*p* = 0.03), and *HOXD10* overexpression (*p* = 0.018) were associated with worse prognosis in GSE53625 dataset. The final multivariate model further confirmed late-stage tumors (*p* = 0.0002; HR = 2.97; 95% CI 1.40–3.12) and *HOXA7* overexpression (*p* = 0.024; HR = 1.58; 95% CI 1.06–2.35) as independent biomarkers of OS in ESCC (Table 4; Figure 3A).

We evaluated the expression profile of *HOXA7* in an independent set of ESCC Brazilian patients, observing *HOXA7* overexpression in ESCC compared to paired NMSM (*p* = 0.016) (Supplementary Figure S3). Furthermore, we performed OS univariate analysis, and *HOXA7* overexpression (*p*-value = 0.0019, HR = 3.29, 95%CI 1.54–6.99) and late-stage tumors (*p*-value = 0.012, HR = 3.02, 95% CI 1.27–7.19) were associated with worse OS rates. Multivariate analysis confirmed that *HOXA7* overexpression is an independent biomarker of worse prognosis in this dataset (*p*-value = 0.039, HR 2.41, 95% CI 1.04–5.56) (Figure 3B; Table 4).

## 4. Discussion

In the present study, we investigated the mutational and expression profiles of *HOX* genes in esophageal squamous cell carcinoma (ESCC). We identified the signaling pathways associated with the putative targets of *HOX* genes overexpressed in ESCC. Our findings demonstrate that *HOXA7* overexpression is an independent biomarker for poor prognosis in this malignancy.

The low frequency of somatic genetic alterations in *HOX* genes observed in ESCC (0–8%) is consistent with other cancers characterized by similar risk factors, such as alcohol consumption and tobacco smoking. For example, 4% of cases of lung squamous carcinoma exhibit copy number alterations or missense mutations in *HOX* genes [29,30]. In comparison, 6% of head and neck tumors show somatic alterations at the HOXD *locus* [30]. These data suggest that somatic mutations are not the primary mechanism underlying the dysregulation of *HOX* genes in tumorigenesis, underscoring the importance of regulatory mechanisms governing *HOX* gene expression in cancer.

We analyzed two transcriptomic datasets, comparing paired ESCC and NMSM, and identified seven *HOX* genes differentially expressed in ESCC. Furthermore, we validated the *HOXA7* expression profile using an independent sample set. Previous studies have attempted to evaluate the expression profiles of *HOX* genes in ESCC using the methodologies available then. Chen and collaborators [11] assessed the expression of *HOX* genes in paired ESCC and NMSM samples from Chinese patients using non-quantitative RT-PCR without adjustment for multiple comparisons. They reported a higher number of deregulated *HOX* genes in ESCC, with nine genes exclusively expressed in tumors (*HOXA10*, *HOXA13*, *HOXB7*, *HOXC4*, *HOXC8*, *HOXD9*, *HOXD10*, and *HOXD13*) and three overexpressed in ESCC relative to NMSM (*HOXA7*, *HOXA9*, and *HOXC6*). Similarly, using quantitative RT-PCR, Takahashi and collaborators [13] observed that 24 of 39 *HOX* genes were overexpressed in ESCC compared to paired NMSM from Japanese patients. Again, they did not use adjustment methods for multiple comparisons. Therefore, the available studies confirm that a subset of *HOX* genes is overexpressed in ESCC, and the technique and statistical criteria applied could explain the discrepancies between our data and other studies.

In addition to the dysregulation of *HOX* genes, we identified putative *HOX* transcriptional targets using in silico analyses and assessed their differential expression in ESCC. Gene-set enrichment analyses highlighted various signaling pathways previously associated with the natural history of ESCC. Notably, in the PI3K-AKT signaling pathway, molecular alterations in PIK3CA and PIK3R3 have been linked to poor prognosis [17,18]. Furthermore, epithelial–mesenchymal transition is recognized as a relevant event for ESCC carcinogenesis, and the aberrant activation of the WNT signaling pathway is also associated with ESCC prognosis [31,32]. This study also identified the enrichment of signaling pathways related to IL-6-JAK, inflammatory responses, and apoptosis. We previously demonstrated that IL-6 mRNA and protein expression are upregulated in ESCC compared to NMSM, with IL-6 overexpression playing a crucial role in ESCC carcinogenesis by promoting anti-apoptotic signaling via *BCL3* overexpression [33].

The dysregulation of *HOXA7* in ESCC is associated with a poorer prognosis, echoing findings from mixed-lineage leukemia, where *HOXA7* overexpression correlates with lower survival rates [34]. Additionally, *HOXA7* has been reported to be overexpressed in colorectal cancer [35], which is also linked to poor prognoses. Similar to our results, which show higher *HOXA7* expression in ESCC mucosa compared to NMSM samples, oral tumors exhibit elevated *HOXA7* levels compared to the dysplastic oral mucosa [36]. This altered expression profile suggests that *HOXA7* could be a central player in the carcinogenesis of the upper aerodigestive tract.

*HOXA7* promotes hepatocellular carcinoma through cyclin E1/CDK2, inducing proliferation, migration, colony formation, and tumorigenesis in vivo [37]. In oral squamous cell carcinoma, *HOXA7* overexpression is linked to aggressive markers such as tumor size, high-grade tumors, vascular and perineural invasion, and lymph node and distant metastases [38]. In ESCC, it has been shown that *HOXA7* induces tumor-associated macrophage infiltration and M2 polarization by promoting CCL2 secretion. Additionally, macrophage secretion of EGF induced by CCL2 promotes ESCC tumor growth [12]. *HOXA7* can also induce *EGFR* expression in ESCC cells [39] and in granuloma cells [39].

The dysregulation of *HOX* genes is a multifactorial process mediated by temporospatial deregulation, gene dominance, and epigenetic perturbations [2]. Epigenetic control, mainly through DNA methylation mediated by DNA methyltransferases/ten-eleven translocations (DNMTs/TETs) regulation of DNA methylation, as well as histone methylation and long non-coding RNA [40], emerges as a crucial mechanism governing *HOX* genes. In breast cancer, TET1 has been shown to impair *HOXA7* function by modulating HOXA promoters, thereby promoting breast tumor growth and metastasis [41]. Recent studies have illuminated the significant role of *HOX*-related non-coding RNAs in modulating chromatin dynamics and gene expression, with implications for the progression of oral squamous cell carcinoma. The authors showed that miR196a was upregulated in oral squamous cell carcinoma samples from patients undergoing surgery and radiotherapy, suggesting its interaction with *HOXA7* and a potential tumorigenic role [36].

This study has limitations, such as the lack of assessment of *HOXA7* protein levels. Nevertheless, we applied stringent statistical criteria and validated the association between *HOXA7* expression and the prognosis of ESCC patients in independent sample sets.

## 5. Conclusions

In conclusion, our findings underscore the rarity of somatic alterations in *HOX* genes in ESCC and shed light on the pivotal role of altered gene expression in driving their dysregulation. Furthermore, the *HOXA7* overexpression emerges as an independent biomarker of poor prognosis in ESCC, providing valuable insights into the disease.

## Figures and Tables

**Table 1:** ESCC patient’s features.

Feature	Variable	Brazilian ESCC Patients
Age—Median (range)		59 (39–77)
Gender	Female	8 (19.51%)
	Male	33 (80.49%)
Tobacco smoking	No	4 (9.76%)
	Yes	35 (85.36%)
	NA	2 (4.88%)
Alcohol drinking	No	5 (12.20%)
	Yes	35 (85.36%)
	NA	1 (2.44%)
Esophagel tumor subsite	Upper	5 (12.20%)
	Middle	11 (26.83%)
	Lower	3 (7.3%)
	More than one subsite	22 (53.66%)
Tumor grade	G2	34 (82.93%)
	G3	7 (17.07%)
Stage	I-II	12 (29.27%)
	III-IV	25 (60.97%)
	NA	4 (9.76%)
Lymph node metastasis	No	8 (19.51%)
	Yes	17 (41.46%)
	NA	16 (39.03%)
Distant metastasis	No	11 (26.83%)
	Yes	17 (41.46%)
	NA	13 (31.71%)

NA: not available.

**Table 2:** ESCC genes differentially expression in two datasets.

Gene Symbol	GSE75241			GSE53625		
	Probe ID	Fold Change (ESCC/NMSM)	*p* Value	Probe ID	Fold Change (ESCC/NMSM)	*p* Value
*HOXA2*	3042756	−1.67	<0.001	CB_015587	−1.58	<0.001
*HOXA7*	3042881	1.50	0.002	CB_015737	2.13	<0.001
*HOXB13*	3761538	2.12	<0.001	CB_015218	2.87	<0.001
*HOXC9*	3416344	1.57	<0.001	CB_015738	3.43	<0.001
*HOXC10*	3416290	2.32	<0.001	CB_019037	10.56	<0.001
*HOXC13*	3416256	1.66	<0.001	CB_019038	15.45	<0.001
*HOXD10*	2516834	4.17	<0.001	CB_011132	4.44	<0.001

**Table 3:** Association analyses between E SCC-overexpressed *HOX* genes and clinicopathological features.

		Frequency		*HOXA2*		*HOXA7*		*HOXB13*		*HOXC9*		*HOXC10*		*HOXC13*		*HOXD10*	
Feature	Variable	n	%	Median	*p* Value	Median	*p* Value	Median	*p* Value	Median	*p* Value	Median	*p* Value	Median	*p* Value	Median	*p* Value
Age	<60	89	49.70%	10.54	0.11	12.94	0.22	5.76	0.026	10.70	0.16	9.59	0.85	9.14	0.38	11.89	0.92
	≥60	90	50.30%	10.83		12.87		6.83		10.91		9.64		9.18		11.88	
Sex	Male	146	81.60%	10.78	0.24	12.95	0.12	6.53	0.78	10.83	0.50	10.14	0.88	9.13	0.67	11.97	**0.009**
	Female	33	18.40%	10.61		12.73		6.15		10.7		9.95		9.24		11.50	
Alcohol Drinking	No	73	40.80%	10.77	0.65	12.74	0.09	6.24	0.73	10.65	0.09	9.94	0.66	8.98	0.06	11.80	**0.01**
	Yes	106	59.20%	10.73		13.04		6.32		10.91		10.22		9.43		12.11	
Tobacco Smoking	No	65	36.30%	10.73	0.8	12.82	0.33	5.84	0.41	10.53	**0.005**	9.99	0.92	9.05	0.27	11.88	0.53
	Yes	114	63.70%	10.76		12.97		6.82		10.97		10.16		9.27		12.00	
Esophageal Tumor Subsite	Upper	20	11.20%	10.96	0.81	13.34	0.09	6.87	0.62	11.23	0.36	9.88	0.70	8.88	0.3	11.76	0.48
	Middle	97	54.20%	10.62		12.98		6.69		10.96		10.00		9.09		12.06	
	Lower	62	34.60%	10.62		12.66		6.87		10.55		10.39		9.43		11.81	
Tumor Differentiation	Well	32	17.90%	10.59	0.68	13.11	0.74	5.66	0.0501	10.56	0.0067 *	9.89	0.47	9.48	0.92	11.8	0.75
	Moderate	98	54.70%	10.61		12.89		6.32		10.72		9.94		9.15		12.03	
	Poorly	49	27.40%	10.79		13.03		7.36		11.27		10.72		9.08		12.05	
Lymph node metastasis	**No**	83	46.40%	10.68	0.55	12.83	0.74	6.87	0.31	10.80	0.89	10.00	0.72	9.06	0.30	11.95	0.95
	Yes	96	53.60%	10.62		12.95		5.73		10.82		10.25		9.40		11.97	
T (TNM)	T1/T2	39	21.70%	10.54	0.3	12.83	0.82	6.35	0.65	11.01	0.16	10.34	0.65	9.40	0.39	11.91	0.84
	T3/T4	140	78.80%	10.66		12.94		6.27		10.75		10.00		9.10		11.88	
Tumor Stage	Early (I/II)	87	48.60%	10.57	0.08	12.82	0.55	6.87	0.09	10.77	0.61	9.63	0.95	9.08	0.37	11.88	0.95
	Late (III)	92	51.40%	10.68		13.01		5.65		10.84		9.61		9.27		12.05	

Legend: * well vs. poorly *p* = 0.008; moderate vs. poorly *p* = 0.029.

**Table 4:** Overall survival analysis.

Feature	Variable	GSE53625								Brazilian Samples							
		Univariate Survival Analysis				Multivariate Analysis				Univariate Survival Analysis				Multivariate Analysis			
		95% CI				95% CI				95% CI				95% CI			
		HR	Low	High	*p* Value	HR	Low	High	*p* Value	HR	Low	High	*p* Value	HR	Low	High	*p* Value
Age	≥60 y vs. <60 y	1.54	1.047	2.26	**0.028**					0.98	0.51	1.89	0.97				
Gender	Male vs. Female	0.78	0.49	1.25	0.3	2.97	1.4	3.12	**0.0002**	0.64	0.27	1.51	0.31				
Stage	I vs. II vs. III	2.15	1.44	3.2	**0.00015**					3.02	1.27	7.19	**0.012**	2.36	0.93	5.96	0.06
Grade	G3/G2 vs. G1	1.35	1	1.82	**0.048**					0.57	0.28	1.17	0.12				
HOXA2	High vs. Low	1.58	1.041	2.408	**0.0316**												
HOXA7	High vs. Low	1.65	1.11	2.45	**0.013**	1.58	1.06	2.35	**0.024**	3.29	1.54	6.99	**0.0019**	2.41	1.04	5.56	**0.039**
HOXB13	High vs. Low	1.9	1.06	3.4	**0.0302**												
HOXC9	High vs. Low	1.52	0.96	2.42	0.073												
HOXC10	High vs. Low	0.28	0.14	1.04	0.061												
HOXC13	High vs. Low	1.28	0.87	1.88	0.13												
HOXD10	High vs. Low	1.3	0.87	1.9	**0.018**												

Bold numbers refer to the significant *p*-value.

## Data Availability

Data are contained within the article or Supplementary Material.

## Supplementary Materials

The following supporting information can be downloaded at: https://www.mdpi.com/article/10.3390/genes15111430/s1, Figure S1: *HOX* genes somatic alteration in ESCC. Oncoprint showed the somatic alterations of the *HOX* genes in esophageal squamous cell carcinoma (ESCC) samples from TCGA. Each row represents one *HOX* gene, and each column represents one tumor sample; Figure S2: Enrichment analyses of differentially expressed HOXA7 gene targets were conducted to identify significant pathways. Using potential targets of differentially expressed *HOXA7* genes in esophageal squamous cell carcinoma (ESCC), this analysis highlighted key signaling pathways in the KEGG (A) and Hallmarks (B) databases. The shades of blue represent the gene count observed within each signaling pathway, with darker blues indicating a higher number of observed genes in each pathway. Figure S3: Validation of *HOXA7* gene expression profile in ESCC. *HOXA7* expression was evaluated by qPCR in esophageal squamous cell carcinoma (ESCC) and paired non-malignant surrounding mucosa (NMSM) samples from the Brazilian population, and significantly higher expression was observed in ESCC samples than in paired NMSM samples (*p* = 0.0168). Min: Minimum; Max: Maximum. Supplementary Table S1: Oligonucleotide sequences. Supplementary Table S2: Potential transcriptional targets of *HOXA2*, *HOXA7*, *HOXB13*, *HOXC9*, *HOXC10*, *HOXC13* and *HOXD10*. Supplementary Table S3: Potential transcriptional targets of *HOXA7*.
