# Supplementary material for: HOXA7 Expression Is an Independent Prognostic Biomarker in Esophageal Squamous Cell Carcinoma
Source: Genes (Basel). 2024 Nov 1;15(11):1430. doi: 10.3390/genes15111430 (PMC11593377; doi:10.3390/genes15111430)
Supplement: Supplementary file 1 [file genes-15-01430-s001.zip › Supplementary Figure 1.pptx]

## Slide 1
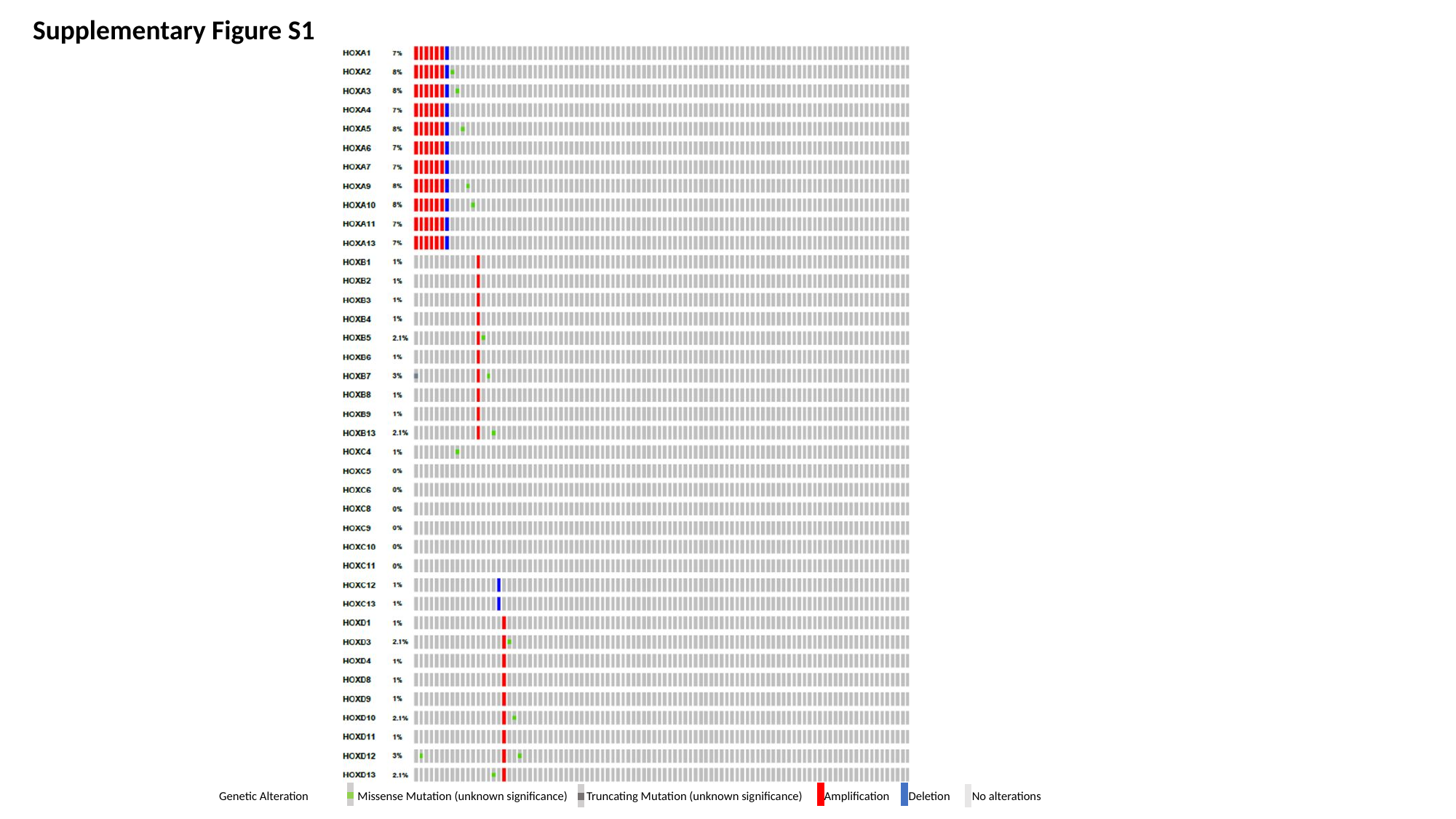

Supplementary Figure S1
Genetic Alteration Missense Mutation (unknown significance) Truncating Mutation (unknown significance) Amplification Deletion No alterations
