# Supplementary figures and images for: HOXA7 Expression Is an Independent Prognostic Biomarker in Esophageal Squamous Cell Carcinoma
Source: Genes (Basel). 2024 Nov 1;15(11):1430. doi: 10.3390/genes15111430 (PMC11593377; doi:10.3390/genes15111430)

## Slide 1
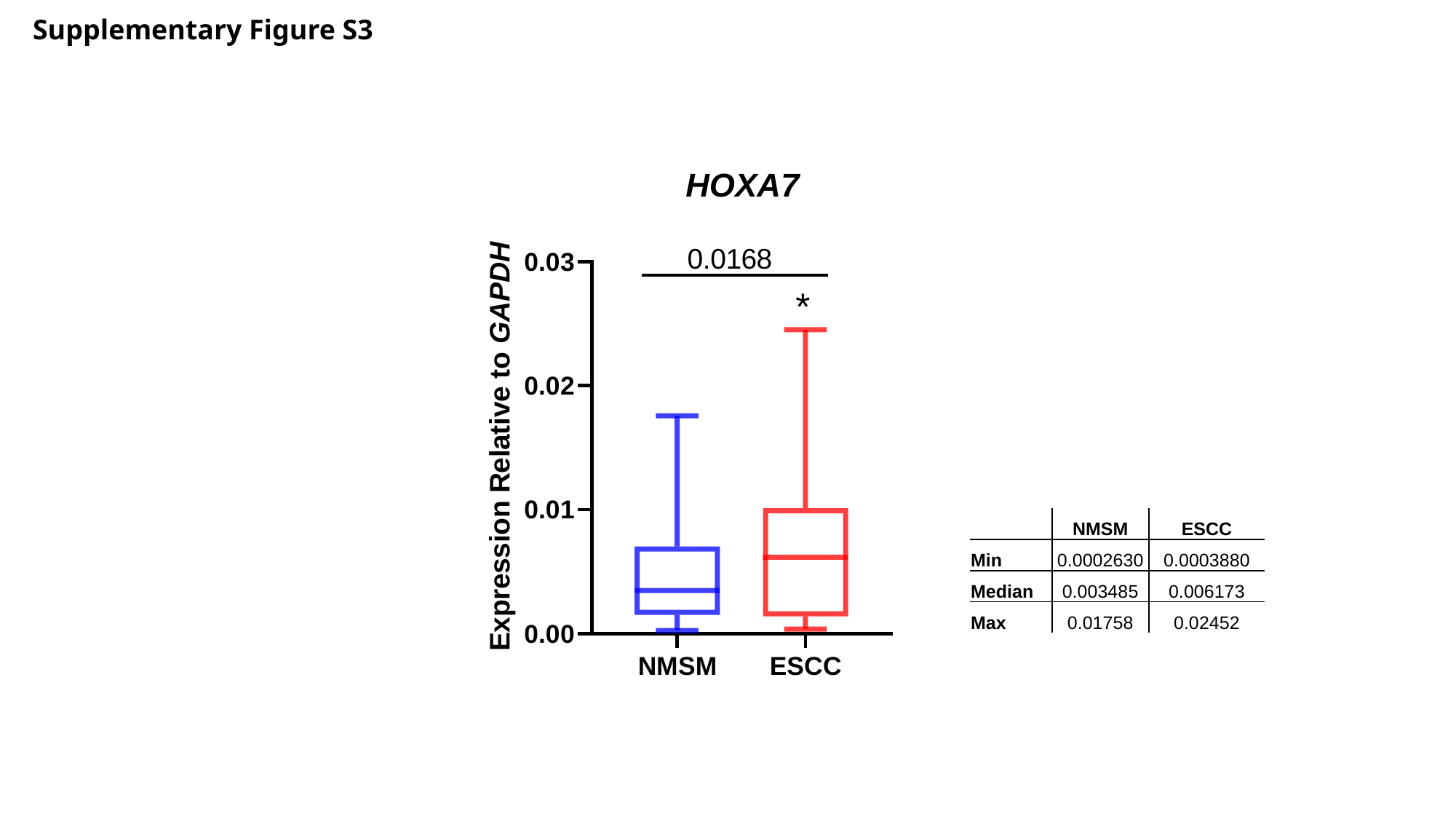

Supplementary Figure S3
| | NMSM | ESCC |
| --- | --- | --- |
| Min | 0.0002630 | 0.0003880 |
| Median | 0.003485 | 0.006173 |
| Max | 0.01758 | 0.02452 |

Supplement: Supplementary file 1 [file genes-15-01430-s001.zip › Supplementary Figure 3-rev.pptx]

## Slide 1
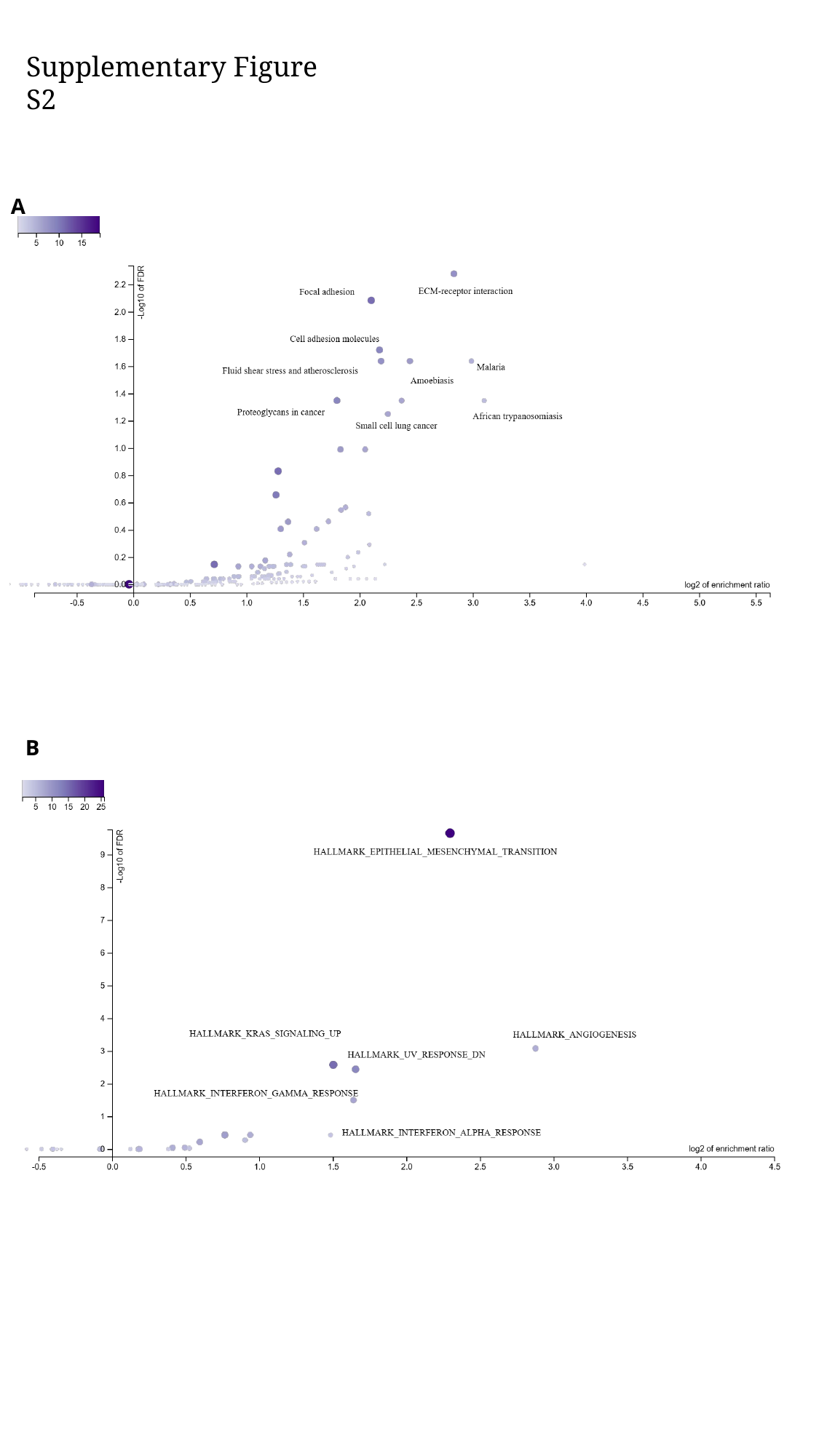

Supplementary Figure S2
A
B

Supplement: Supplementary file 1 [file genes-15-01430-s001.zip › Supplementary_Figure2_rev.pptx]
